# Experimental and Computational Studies to Characterize and Evaluate the Therapeutic Effect of *Albizia lebbeck* (L.) Seeds in Alzheimer’s Disease

**DOI:** 10.3390/medicina55050184

**Published:** 2019-05-21

**Authors:** Uzma Saleem, Zohaib Raza, Fareeha Anwar, Bashir Ahmad, Sundas Hira, Tahir Ali

**Affiliations:** 1 Department of Pharmacology, Faculty of Pharmaceutical Sciences, Government College University, Faisalabad 38000, Pakistan; zuhaib.raza@live.com; 2 Riphah Institute of Pharmaceutical Sciences, Riphah International University, Lahore 54000, Pakistan; fareeha.anwar@riphah.edu.pk (F.A.); sundas.hira@riphah.edu.pk (S.H.); tahir.ali@riphah.edu.pk (T.A.)

**Keywords:** memory loss, antioxidant, neuroprotective, acetylcholinesterase inhibitor, molecular docking

## Abstract

*Background and Objectives*: Alzheimer’s disease (AD) is a neurodegenerative disorder that deteriorates daily life due to loss of memory and cognitive impairment. It is believed that oxidative stress and cholinergic deficit are the leading causes of AD. Disease-modifying therapies for the treatment of AD are a challenging task for this century. The search for natural and synthetic agents has attracted the attention of researchers. The objective of this study was a scientific approach to search for most suitable remedy for AD by exploiting the potential of *Albizia lebbeck* (L.) seeds. *Materials and Methods*: Hydromethanolic extract of *Albizia lebbeck* seeds (ALE) was prepared by maceration. The plant was characterized by physico-chemical, phyto-chemical, and high-performance liquid chromatography (HPLC). Thirty-six Wistar albino rats were used in this study and divided into six groups (*n* = 6). Group I: normal control; Group II: disease control (AlCl_3_; 100 mg/kg); Group III: standard control (galantamine; 0.5 mg/kg); Groups IV–VI were treated ALE at 100, 200 and 300 mg/kg dose levels, respectively. All the treatments were given orally for 21 consecutive days. Y-maze, T-maze, Morris water maze, hole board, and open field behavioral tests were performed to analyze the cognitive impairment. Biochemical, histological, and computational studies were performed to support the results of behavioral tests. *Results*: HPLC analysis indicated the presence of quercetin, gallic acid, m-coumaric acid, and sinapic acid. ALE significantly improved the memory and cognitive impairments. Endogenous antioxidant stress biomarker levels and histopathological outcomes supported the therapeutic potential of *A. lebbeck* in AD. Cholinergic deficits were also ameliorated by ALE co-administration, possibly by the inhibition of hyperactive acetylcholinesterase (AChE). Docking studies supported the potential of ALE against AD. *Conclusions*: The data suggested that ALE has neuroprotective potential that can be exploited for beneficial effects to treat AD.

## 1. Introduction

Alzheimer’s disease (AD) is a neurodegenerative disorder with progressive neuronal loss and dysfunction. It has the clinical manifestations of irreversible decline in memory and deterioration of cognitive functions. AD has accounted for 60–80% of all the cases of dementia and affects approximately 24 million people globally. The prevalence of AD is an age-related phenomenon with a 15-fold increase in the people aged between 65 to 85 [1]. The prevalence of AD is expected to increase as a consequence of growing aging population in both developing and developed countries [2]. The precise etiology of AD pathogenesis is still a challenge to understand, despite decades of extensive research. There has been several hypotheses explaining the pathogenesis of AD as a function of impaired cholinergic transmission, accumulation of amyloidal plaques, neurofibrillary tangles, and oxidative stress [3]. Oxidative stress has been the long-established pathological hallmark of neurodegeneration in AD. It has also been found that accumulation of amyloid plaque induces the redox imbalance that makes oxidative stress a key pathological characteristic of AD [4]. Neuronal circuits are generated by synaptic connections of neurons and are involved in sensory perception, behavior, emotion, and memory. They provide information to neurons as electrical impulses that relay from presynaptic to postsynaptic neurons by the release of neurotransmitters [5]. The role of synapses are dynamic and highly organized and can be changed by oxidative stress. In neurodegenerative diseases such as AD these dynamic circuit connections are functionally altered and, due to this, brain functioning is severely affected. In addition to the normal synaptic functioning, neuronal health is very important. Neurons regulate protein turnover and remove damaged and aggregated proteins, and depend upon electrical signals to direct neuronal survival and health [6]. In addition, impaired cholinergic transmission is another key pathological phenomenon of AD that is regulated by the induction of acetylcholinesterase (AChE) in synapses. The inhibition of AChE has been directly correlated with improvement of cholinergic transmission, and serves as a measure to symptomatically treat severe dementia. So far, AChE is the only target for which the Food and Drug Administration (FDA) has approved drugs to treat the memory deficits in AD [7]. However, the limited therapeutic options, severe adverse effects of current medication, and expanded knowledge of AD pathogenesis demands the development of new safer treatments that address both neurodegeneration and memory deficits in the context of AD.

The plants are one of the most ancient and significant contributors to the development of modern medicinal systems. The knowledge of therapeutic plants is the basis of comprehensive medicinal systems such as Ayurveda and traditional Chinese medicine (TCM). The spread of this knowledge from generation to generation has significantly contributed to the development of therapeutic remedies throughout history. There are 2.5 million species of flowering plants around the world with about half of the species in tropical forests. Interestingly, only 1% of tropical forest plant species have been pharmacologically characterized, which may justify the potential of this kingdom as a source of novel therapeutic compounds [8]. *Albizia lebbeck* (L.) belongs to genus *Albizia* of the Fabaceae family. It is a deciduous tree with bi-pinnate leaves and white fragrant flowers with fruit that is a pod containing six to 12 seeds [9]. Traditionally, the seeds have been used for a variety of therapeutic purposes such as treatments for leukoderma, piles, diarrhea, and gonorrhea, as an aphrodisiac, and as a brain tonic [10]. There is evidence that ethanolic extract of *A. lebbeck* increases the brain content of gamma-amino butyric acid (GABA) and serotonin that is attributed to depressive and anti-convulsive traits [11]. The ethanolic extract of *A. lebbeck* bark has been found to exhibit anti-depression activity with efficacy comparable to imipramine and fluoxetine [12]. Moreover, the neuropharmacological potential of this species has also been corroborated for its sedative and anxiolytic activity [13]. However, there is no direct scientific evidence that suggests the therapeutic potential of *A. lebbeck* seeds in AD. Therefore, the present study is aimed at evaluating the therapeutic potential of *A. lebbeck* seeds in AD.

## 2. Materials and Methods

### 2.1. Chemicals

Methanol, Hydrochloric acid (HCL), Sulfuric acid (H_2_SO_4_), Ethanol, Petroleum ether, Triton-X, Sodium carbonate (Na_2_CO_3_), Sodium Hydroxide (NaOH), Copper sulphate (CuSO_4_), Potassium sodium tartarate (KNaC_4_H_4_O_6_.4H_2_O), Bovine serum albumin (BSA), Gallic acid, Piperine, quercetin, Aluminum nitrate (Al (NO_3_)_3_), 2,2-diphenyl-1-picrylhydrazyl (DPPH), potassium ferricyanide, Aluminum chloride (AlCl_3_), potassium acetate, Trichloroacetic acid (TCA), 5,5’-dithio-bis(2-nitrobenzoic acid) (DTNB), Hydrogen Peroxide (H_2_O_2_), potassium dihydrogen phosphate, Sodium Tartarate, pyragallol, potassium hydroxide, and di-potassium hydrogen phosphate were purchased from Sigma-Aldrich (St. Louis, MO, USA). Ketamine was obtained from Caraway pharmaceutical (Islamabad, Pakistan). Galantamine was obtained from Reko Pharmacia (Lahore, Pakistan). All the chemicals used were of analytical grade.

### 2.2. Collection and Preparation of Plant Material

*Albizia lebbeck* (L.) seeds were collected from botanical garden at the University of Agriculture Faisalabad (UAF). They were identified by the taxonomist Prof Dr Mansoor from the department of botany. Following authentication, a voucher specimen (620-1-18) was deposited in the UAF herbarium. Seeds were washed, air dried, grinded, and sifted into a fine powder.

### 2.3. Physicochemical, Phytochemical and Qualitative Analysis of Seeds Powder Material

The methods listed in United States Pharmacopoeia—National Formulary (2003) were adopted to analyze the physicochemical parameters including moisture content, total ash, acid-insoluble ash, water-insoluble ash, sulphated ash, alcohol-soluble extractives, and water-soluble extractives. Phytochemical analysis was performed to estimate the total protein, lipids, and carbohydrates content [14]. Powder material was qualitatively analyzed by Fourier-transform infrared spectroscopy (FTIR) [14].

### 2.4. Preparation and Characterization of Albizia lebbeck Seeds Extract

Powder material (1 kg) was cold macerated in 1:2 ratio with 80% aqueous methanolic solvent (2 L) for 14 days with 12 h periodic stirring. Finally, macerate was filtered through Whatman No. 1 filter paper and filtrate was concentrated using rotary evaporator under reduced pressure at 40–45 °C and extract yield percentage was calculated as follows:

Extract yield percentage (%) = (weight of pure extract/weight of powder macerated) × 100

Quantitative phytochemical analysis of extract was performed to estimate the primary and secondary metabolites [14]. In vitro antioxidant potential of extract was evaluated by 2,2-diphenyl-1-picrylhydrazyl (DPPH) scavenging and reducing power assay following methods described previously [15]. The identification of metabolites was carried out by the means of a high-performance liquid chromatography (HPLC) system LC-10A (Shimadzu, Nagoya, Japan) using Shim-Pack CLC-ODS C—18 column (25 cm × 4.6 mm, 5 µm) [16]. Mobile Phase contained solvent A (H_2_O: Acetic acid—94:6, pH = 2.27) and B (100% acetonitrile). The isocratic elution of fractions was carried out at flow rate of 0.1 mL/min at 30 °C and detected by ultra-violet (UV)-visible detector at 280 nm wavelength.

### 2.5. Animals and Experimental Design

The experimental studies were performed on adult Wistar albino rats of same age, strain, and sex. Rats were procured from UAF and maintained in the animal house of the department of pharmaceutical sciences, Government College University Faisalabad (GCUF), Faisalabad. All the animals were maintained on a laboratory diet with water ad libitum and acclimatized for the period of one week. Animals were housed in stainless steel cages in a temperature-controlled environment (24 ± 1 °C) with natural light and dark cycles and free from chemical contamination. All the animals were provided with personalized human care. The animal observations in experiments were carried out under room temperature in a noiseless facility with an adequate light system. The experimental study (No. 19589) was performed after getting ethical approval dated 06/09/2018 from the Institutional Review Board with reference no. GCUF/ERC/1989 ruled under the regulation of Institute of Laboratory Animal Resources, Commission on Life Sciences University, National Research Council (1996).

The following experimental design was established to evaluate the in-vivo anti-AD potential of of *Albizia lebbeck* seeds extract (ALE).

(a) Group I: Normal control (NC);
(b) Group II: Disease control (AlCl_3_);
(c) Group III: Standard control (STD);
(d) Group IV: 100 mg/kg treatment control (ALE 100);
(e) Group V: 200 mg/kg treatment control (ALE 200);
(f) Group VI: 300 mg/kg treatment control (ALE 300).

There were six rats (*n* = 6) weighing 100–150 g in each group.

The NC (Group I) received the distilled water as vehicle. Aluminum chloride (100 mg/kg, p.o.) was administered to Groups II–VI to induce the AD in these groups. The standard control (Group III) was administered with galantamine (0.8 mg/kg, p.o.) as the standard treatment. The treatment Groups IV–VI received the 100, 200, and 300 mg/kg oral dose of aqueous methanolic ALE dissolved in distilled water. All the treatments were given via oral route once daily for consecutive 21 days. After 21 days of treatment, rats were subjected to behavioral, biochemical, and histological experiments.

#### 2.5.1. Behavioral Experiments

##### Morris Water Maze Test

The study was performed with some modifications to assess the induction of AD and cognitive deficits in experimental rats following the treatment [17]. The apparatus was a large plastic circular swimming pool with 59 cm and 45 cm diameter at circumference and bottom, respectively. The depth of the pool was 32 cm, filled with water (24 ± 1 °C), and 500 mL of milk up to 20 cm of the pool length. The pool was divided into four equal hypothetical quadrants and a hidden platform (W 8 cm × H 16 cm) was in one quadrant. The experiment was performed in three phases spanning 6 days. The first day of behavioral study was dedicated to swimming training of each rat for 1 min in the absence of the hidden platform, which made up the first phase of experiment. The second phase consisted of daily training trial sessions for each rat for four consecutive days in the presence of a hidden platform in the pool. During this phase, rats could swim for 2 min to locate the hidden platform and stay there for 10 s; time latency to locate the hidden platform served as a measurement of development of spatial learning and memory in rats (i.e., acquisition latency). If animals were unable to locate the hidden platform then they were placed on it and allowed to stay there for 10 s. The probe trial session was performed on the last day of experiment (i.e., the third phase, in which animals could swim for 2 min in the absence of a hidden platform to search for it. The swimming time was recorded for the quadrant where the platform was placed earlier.

##### Open Field Test

This study was performed to assess the exploratory behavior of animals [18]. The apparatus for this study was a wooden square box (W 100 cm × D 100 cm × H 45 cm) covered with resin and a floor divided into 25 squares. Rats were allowed to move freely into the box for two minutes and the following parameters were observed and recorded: number of squares (both central and peripheral) explored (i.e., horizontal exploration); number of rearing (i.e., vertical exploration) [18].

##### Hole Board Test

The test was performed according to the methods adopted in the literature [19]. The apparatus is made up of a plastic chamber (L 30 cm × W 30 cm) with 16 holes, each evenly distributed at the floor of chamber. This test employs the principle of an animal’s head-dipping behavior as a measurement of escaping and of an exploratory tendency. The rats are placed in the chamber for 120 s and the number of head dips, into the holes, is recorded for each animal.

##### Y-Maze Test

The test was performed according to the method described in the literature [20]. The Y-maze test evaluates the animal’s spatial learning and memory based on spontaneous alternation. Spontaneous alternation is defined as behavior of successive entries to the arms of the maze. The apparatus is made of three arms (L 45 cm × H 13 cm × W 11 cm) attached to each other at an angle of 120°. The rats were placed in open arms and allowed to navigate the maze for 120 s, and a percentage alternation is calculated as a measure of cognition for spatial learning and working memory. Percentage alternation was calculated by the following formula:

% Alternation: (Actual alternation / maximum spontaneous alternation) × 100

Where: Maximum spontaneous alternation = Total number of arms entered −2

##### T-Maze Test

This test evaluates the spatial learning and memory retention in rats by the method described previously [21]. The T-shaped apparatus has two goal arms attached to the stem. The test is performed in two phases, the spontaneous alternation and rewarding alternation. The two trials were performed in quick succession for each animal. The spontaneous alternation is the rat’s behavior that it does not tend to enter the arm visited in the earlier trial. The spontaneous alternation trial is followed by the rewarding trial after 24 h of starvation to the animal. The animal is rewarded with food if it alternates on the rewarding trial, i.e., rewarding alternation. Each trial was carried out for 120 s of observation.

After the behavioral experiments, the animals were anesthetized with intramuscular injection of ketamine hydrochloride (24 mg/kg). Following anesthetization, the animals were sacrificed by decapitation to excise the brain tissue, and the carcass was buried. The excised brain tissues were weighed and stored frozen at −80 °C for further use in histological and biochemical experiments.

#### 2.5.2. Biochemical Experiments

##### Estimation of Oxidative Stress

The method of Hira et al. was adopted to estimate the biochemical markers of oxidative stress in the brain [22]. The excised brain tissues were homogenized in ratio 1:10 (*w*/*v*) with phosphate buffer (7.4 pH) in tissue homogenizer. The tissue homogenates were centrifuged in 600 rpm for 10 min at 4 °C. Following centrifugation, the clear supernatant was obtained to evaluate the following biochemical parameters and their correlation to the oxidative stress in brain.

i. Estimation of glutathione content:

The supernatant of tissue homogenate (1 mL) was further precipitated with 10% TCA (1 mL), and aliquot obtained was combined with phosphate solution (4mL) and 0.5 mL DTNB reagent. This solution was further analyzed with UV spectroscopy to measure the absorbance at 412 nm for determination of glutathione (GSH) content by the following formula:

GSH = Y − 0.00314 / 0.034 × DF ÷ BT × VU

where DF = dilution factor (i.e., 1); VU = volume of aliquot; Y = absorbance at 412 nm; BT = tissue homogenate of brain (1 mL).

ii. Estimation of superoxide dismutase activity:

Tissue homogenate supernatant (0.1 mL) was instilled with 2.8 mL of 0.1 M potassium phosphate buffer (pH 7.4) and pyrogallol solution (0.1 mL) that provided the 3 mL of solution. The solution was analyzed with UV spectroscopy at 325 nm. Different concentrations (10 µL–100 µL) of SOD were used to plot its standard curve. The activity of superoxide dismutase (SOD) was measured by the following regression equation:

Y = 0.0095x + 0.1939

iii. Estimation of Catalase activity:

Tissue homogenate supernatant (0.05 mL) was combined with 50 mM phosphate buffer (pH 7, 1.95 mL) and 30 mM hydrogen peroxide (1 mL) to make a reaction mixture. The resultant mixture was used for UV spectroscopic analysis at 240 nm. Catalase (CAT) activity was calculated by the following formula:

CAT activity = δOD / E × volume of sample (mL) × mg. of protein

where: δOD = changing absorbance/minute; E = extinction coefficient of hydrogen peroxide (i.e., 0.071 mmol cm^−1^).

Difference concentrations of BSA were used to plot the protein standard curve. Protein content was calculated by the following regression equation:

Y = 0.00007571x + 0.0000476

##### Measurement of Acetylcholinesterase Activity

Esterase activity was measured by the method described earlier [23]. Supernatant of brain tissue (0.4 mL) was added into 2.6 mL of phosphate buffer (8 pH, 0.1 molar) in the cuvette. It was further followed by the addition of 100 µL DTNB reagents and 20 µL of acetylthiocholine iodide. Thiocholine reacts with the DTNB and produces the yellow color that is measured at 412 nm. The following equation is used to calculate the AChE activity:

R = 5.74 × 10^−4^ × A/CO

where R = rate in moles of substrate hydrolyzed/min/gm of tissue; A = absorbance at 412nm; CO = original concentration of brain tissue (mg/mL).

#### 2.5.3. Histological Experiment:

Excised brain tissue was fixed in 4% paraformaldehyde, embedded in paraffin and sliced into 5 µm sections [18]. These sections were stained with hematoxylin and eosin (H&E) and examined under light microscopy.

##### Acute Oral Toxicity

Acute oral toxicity studies were carried out according to Organization of Economic Corporation and Development (OECD) guidelines. Animals were treated with the 2000 mg/kg dose via oral route in a single dose. Mortality was observed initially for the first 48 h till the 14th day [24].

#### 2.5.4. Molecular Docking

**Figure 1:**
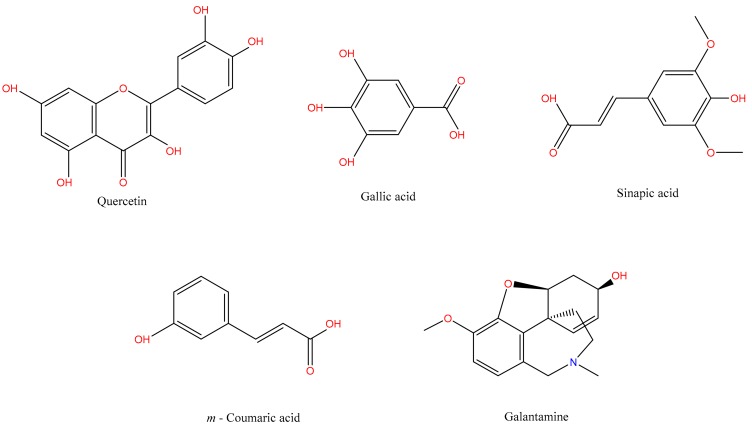
Chemical structures of ligands docked against Acetylcholinesterase (AChE).

The identified phytochemicals were in-silico modeled to predict their activity for AChE by docking function using Autodock Vina 1.1.2 [25,26]. Three dimensional (3D) conformers of quercetin (CID: 5280343), Gallic acid (CID: 370), Sinapic acid (CID: 637775), *m*-Coumaric acid (CID: 637541),and Galantamine (CID: 9651) were retrieved from PubChem database and converted into PDB (Protein Data Bank) formats (Figure 1).

The 3D crystallized PDB structure of AChE (PDB ID: 1J06) was retrieved from RSCB Protein Data Bank (Research Collaboratory for Structural Bioinformatics; http://www.rscb.org/). The rigid protein and flexible ligands were prepared and converted into PDBQT (Protein Data Bank, Partial Charge (Q) & Atom Type (T)) format using Autodock Tools 1.5.6. Gasteiger charge was assigned to each ligand. The hydrogen bonds were added to correct the tautomeric states and ionization of protein residues and non-polar hydrogens were merged. The grid box size was set to 48 × 40 × 32 (x,y,z) dimensions for search space with default grid point spacing (i.e., 0.375 Å) and centered to 32.473 × 20.287 × 10.477 (x,y,z) coordinates. Binding energy (Kcal/mol) of each ligand, to AChE, was calculated by scoring function of Autodock Vina 1.1.2. The binding pose with lowest binding energy was considered and inhibition constant (Ki) was calculated [27]. The binding energy and Ki of galantamine was considered to be the standard’s threshold. The interactions of ligand-target complex were visualized by the Accelrys discovery studio visualizer v17.2.

### 2.6. Statistical Analysis

The experimental results were expressed in mean ±SEM, *n* = 6. GraphPad Prism version 5.0 was used to statistically analyze the intergroup variations by one-way and two-way ANOVA followed by Tukey multiple comparison and Bonferroni post-tests, respectively. The results with *P* < 0.05 were considered statistically significant.

## 3. Results

### 3.1. Physicochemical, Phytochemical, and Qualitative Analysis of Powder Material

Moisture content of the powder material was found to be within the recommended hygroscopicity limits that ensured the material is devoid of microbial contamination. The analysis of ash content showed the acceptable inorganic adulteration [28]. The inorganic impurities were found to be highly insoluble in water. In addition, significant values of water- and alcohol-soluble extractives indicated the aqueous-alcoholic cosolvent with higher extractive capacity and purity. The result of these typical parameters is tabulated (Table 1).

**Figure 2:**
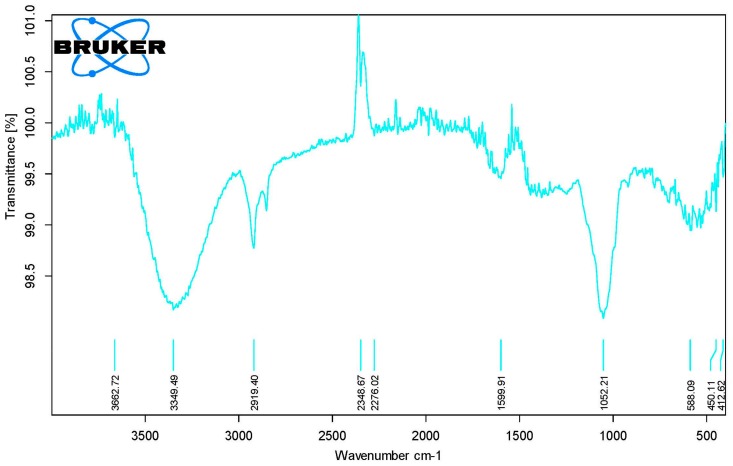
Fourier-transform infrared spectroscopy (FTIR) spectrum of pulverized seeds of the *Albizia lebbeck* (L.).

The phytochemical analysis of crude powder indicated the carbohydrate-based components constituted the 49% of phytochemicals. The phytochemical fraction of total protein and lipids was found to be 2.272% and 0.27%, respectively. Qualitatively standardization of crude powder fingerprinted the phytochemicals with typical IR spectral peaks of N-H (3662.72 cm^−1^, 3349.49 cm^−1^), O-H (2919.4 cm^−1^), C≡N (2348.67 cm^−1^, 2276.02 cm^−1^), Aromatic C=C (1599.91 cm^−1^), C-F (1052.21 cm^−1^), C-Br (588.09 cm^−1^) and C-I (450.11 cm^−1^, 412.62 cm^−1^) functional groups (Figure 2). Spectral analysis revealed the probable presence of alkaloids, polyphenolics, flavonoids and halogenated hydrocarbons [29].

### 3.2. Preparation and Characterization of ALE

Aqueous methanolic ALE produced the yield of 9.9% *w*/*w* with respect to the amount of powder material macerated. The quantitative phytochemical analysis of ALE showed the total glycosaponins (68.033 ± 0.606%) constitutes the major fraction of primary metabolites as compare to the total proteins (2.277 ± 0.007%) (Table 2). The result was consistent with the higher fraction of acidic polyphenolics (88.6 ± 0.032%) and flavonoids (36.327 ± 0.049%) as compared to basic alkaloidal (1.27 ± 0.002%) secondary metabolites.

**Figure 3:**
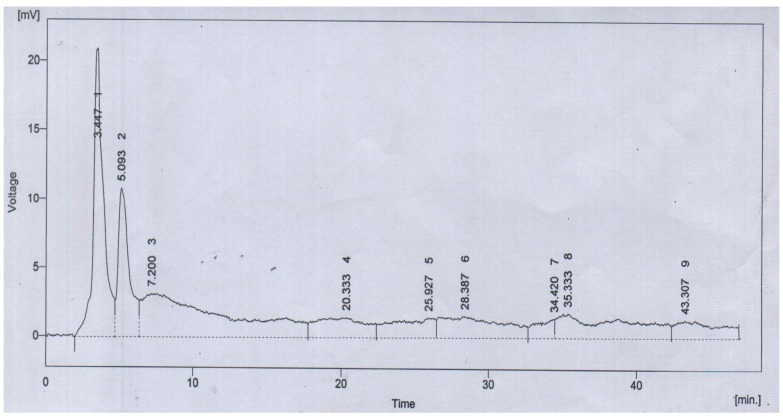
HPLC-UV chromatogram of *Albizia lebbeck* (L.) seeds extract (ALE).

The antioxidant capacity of ALE was manifested as the significant DPPH free-radical scavenging capacity of 86.1 ± 0.6% inhibition with reference to ascorbic acid as a standard. The reducing power potential was comparable and found to be 1.6 ± 0.3 mmol/g equivalents of ascorbic acid. The HPLC chromatogram of ALE resolved the four major peaks with retention time of 3.447 (1), 5.093 (2), 20.333 (4) and 25.927 (5) min (Figure 3).

These peaks were characterized with polyphenolics and flavonoid standards. The compounds were identified as quercetin (1), gallic acid (2), *m*-coumaric acid (4) and sinapic acid (5) (Table 3).

### 3.3. Behavioral Experiments

#### 3.3.1. Morris Water Maze Test

**Figure 4:**
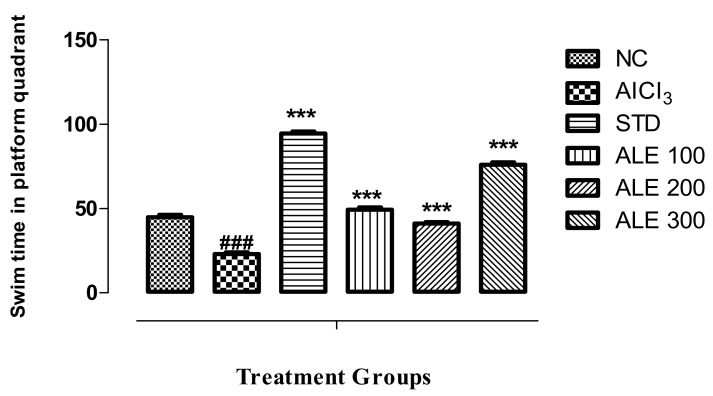
Swim time of the group in the quadrant of platform (previously placed) in Morris water maze test; values are expressed as mean ± SEM, *n* = 6. The intergroup variation was measure by Prism one-way ANOVA followed by Tukey multiple comparison post hoc test, *** *P* < 0.001 was given in comparison to the AlCl_3_ treated group. ^###^ *P* ≤ 0.001 was given in comparison to normal control.

In the probe trial session, the rats will be in search of the quadrant that reflects spatial memory and cognition. During this phase, the swim time in the quadrant of platform (previously placed) is inversely proportional to the deficits in memory. The AlCl_3_-treated rats exhibit marked reduction in swim time (Figure 4). On the other hand, STD group revealed the highest spatial learning and memory as compared to all the groups, which also depicts the highly significant variation (*P* < 0.001) as compared to the AlCl_3_ treated group. However, the recovery from memory deficits was also highly significant (*P* < 0.001) as compared to the AlCl_3_ group. Among the *A. lebbeck*-treated groups (ALE 100, ALE 200, ALE 300), only ALE 300 has the comparable efficacy to improve the cognitive abilities (i.e., spatial learning or memory).

#### 3.3.2. Open Field Test

**Figure 5:**
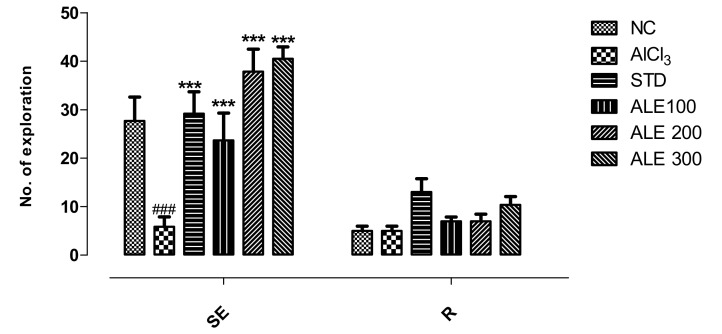
Horizontal exploration (SE) and rearing (R) among groups in open field test; values are expressed as mean ±SEM, *n* = 6. The intergroup variation was measured by prism two-way ANOVA followed by Bonferroni post hoc test. *** *P* < 0.001 was given in comparison to AlCl_3_ group. ^###^ *P* ≤ 0.001 was given in comparison to normal control.

The AlCl_3_ group revealed significant reduction in horizontal explorations (squares crossing) but comparable vertical explorations (rearing) to NC group. Decline in horizontal square crossings and rearing is directly proportional to poor locomotor and cognitive abilities. The ALE 200 and ALE 300 groups revealed the highest number of horizontal explorations as compared to all other groups. The variation of these groups was highly significant (*P* < 0.001) as compared to AlCl_3_ group. Although the STD group revealed highly significant (*P* < 0.001) improvements in horizontal exploration, this fraction was lower than that of the ALE 200 and 300 groups, and comparable to the NC group. However, rearing was not found to be significantly different among all the groups (Figure 5).

#### 3.3.3. Hole Board Test

**Figure 6:**
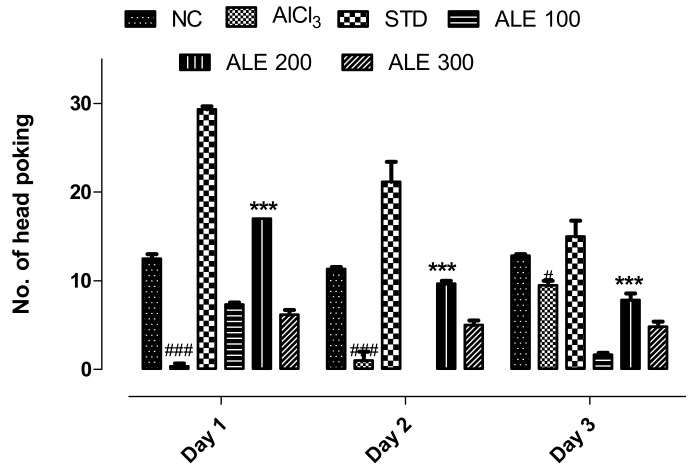
Evaluation of escaping tendency and memory retention among the groups by hole board test; values are expressed as mean ±SEM, *n* = 6. The intergroup variation was measure by prism two-way ANOVA followed by Bonferroni post hoc test. *** *P* < 0.001 was given in comparison to the AlCl_3_ group. ^###^ *P* ≤ 0.001 was given in comparison to normal control.

The AlCl_3_-treated group revealed the lowest number of hole-dipping as compared to all other groups. The highest number (30) of poking was observed in the STD group that was significantly (*P* < 0.001) different from that of the AlCl_3_ group (Figure 6). The STD group revealed the declined in the frequency of head-dipping throughout the duration of study that reflected memory retention as compared to the opposite behavior of AlCl_3_. The ALE 200 and ALE 100 groups also showed the same pattern of poking and memory retention, but with relatively lower pokes as compared to STD group. However, the frequency of head-dipping of ALE 200 declined significantly (*P* < 0.001) as compared to the AlCl_3_ group.

#### 3.3.4. Y-Maze Test

**Figure 7:**
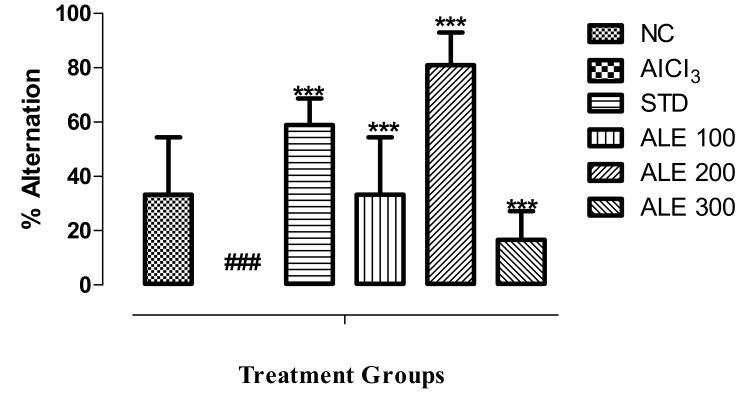
Percentage alternation of behavior among the groups in Y-maze test; values are expressed as mean ±SEM, *n* = 6. The intergroup variation was measure by prism one-way ANOVA followed by Tukey multiple comparison post hoc test. *** *P* < 0.001 was given in comparison to AlCl_3_ group. ^###^ *P* ≤ 0.001 was given in comparison to normal control.

The percentage alternation is the function of spatial learning, memory retention, and cognitive abilities. The rats in AlCl_3_ group revealed 0% alternation, reflecting severe decline in spatial learning and memory retention. On the other hand, the STD group was found to have significantly higher % alternation (Figure 7). However, the % alternation in ALE 200 group was highest (*P* < 0.001) as compared to all other groups. The ALE 100 group also provided a moderate percentage of alternation as compared to ALE200. Surprisingly, the ALE 200 group was found to have the significantly different percentage of alternation as compared to ALE 300 (*P* < 0.005).

#### 3.3.5. T-Maze Test

**Figure 8:**
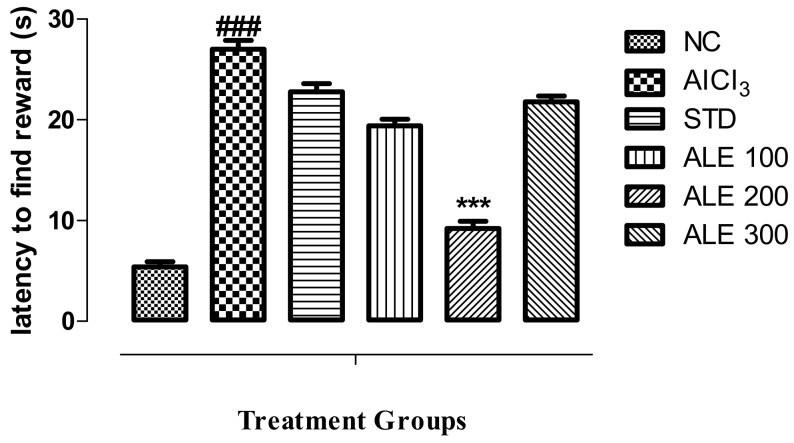
Latency (s) to find the reward among the groups in T-maze; values are expressed as mean ±SEM, *n* = 6. The intergroup variation was measure by prism one-way ANOVA followed by Tukey multiple comparison post hoc test. *** *P* < 0.001 was given in comparison to the AlCl_3_ treated group. ^###^ *P* ≤ 0.001 was given in comparison to normal control.

Time latency to find the reward is the function of decline in memory and cognition. The AlCl_3_ group revealed the highest time latency to find the reward as compared to all other groups (*P* < 0.001) (Figure 8). The time latency of STD, ALE 100, and ALE 300 has comparable values. However, the ALE 200 has been found to exhibit the lowest time latency as compared to all other groups, thereby reflecting higher abilities of cognition as compared to all other groups (*P* < 0.001).

### 3.4. Biochemical Experiment

#### 3.4.1. Estimation of Oxidative Stress

Endogenous antioxidant capacity of rat brain tissue was assessed to measure the status of oxidative stress following induction of AD by AlCl_3_ administration. The content of GSH and antioxidant enzyme activities in brain tissue is directly related to the development of oxidative stress in AD. These parameters were found to be saturated or severely declined in the AlCl_3_ group, which reflects the oxidative stress (Table 4). The dose-dependent recovery of enzymatic activity and GSH content was observed in treatment controls (ALE groups). The recovery of SOD activity and GSH content was highly significant (*P* < 0.001) among all the treatment groups, including the STD group. These parameters were found to be severely compromised in the AlCl_3_ treated group. However, the pattern of CAT activity recovery was not the same in all treatment groups. The highest recovery of CAT activity was observed in ALE 300 (*P* < 0.001), while ALE 100 and ALE 200 groups were found to recover CAT activity comparable to STD (*P* < 0.005).

#### 3.4.2. Assay of Tissue Acetylcholinesterase Activity

**Figure 9:**
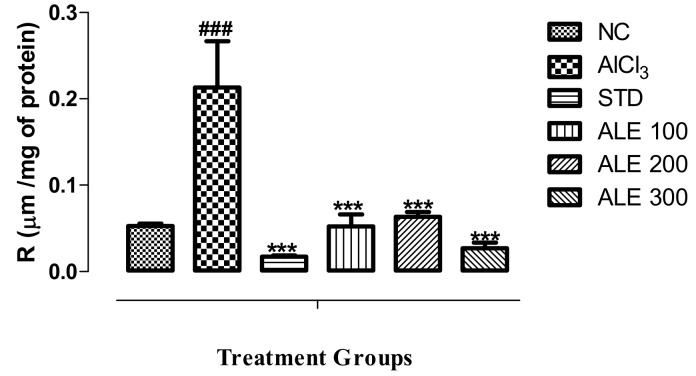
Assay of AChE activity in brain tissue among treated groups; values are expressed as mean ±SEM, *n* = 6. *** *P* < 0.001 in comparison to the AlCl_3_ group. ^###^ *P* ≤ 0.001 was given in comparison to normal control.

The highest enzymatic activity was observed in the brain tissues of the AlCl_3_ group. There was significant (*P* < 0.001) decline in the activity of enzyme in STD group as compared to AlCl_3_ group (Figure 9). However, this pattern was also followed by the ALE 300 group that also significantly (*P* < 0.001) inhibited the enzymatic activity that is comparable to the STD group. The ALE 300 group also showed relative inhibition to the ALE 100 group. However, there were non-significant variations in the activities ALE groups as compared to NC as depicted by the relative titer of R.

### 3.5. Histological Experiments

**Figure 10:**
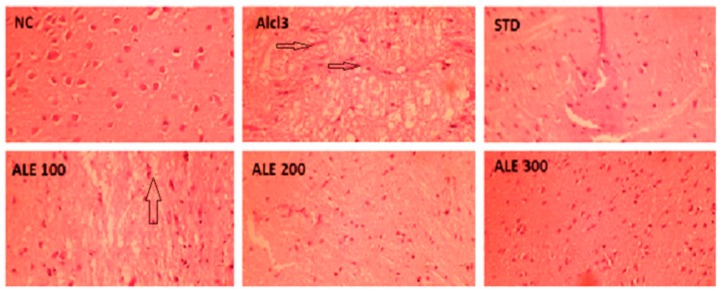
Histopathological changes in the cerebral cortex of experimental groups. Arrows show neurofibrillary tangles.

Histological examination of NC brain tissue stained with H&E revealed the active neuronal cells with normal structure, outlines, and chromatin content. The brain tissue of AlCl_3_ showed the severe histopathological manifestations with marked neurodegeneration, vacuolization (i.e., lipid peroxidation) and neurofibrillary tangles (long pink cytoplasmic filaments). These manifestations were significantly ameliorated in the STD group with appearance more or less like NC tissue sections. The neuronal degeneration and neurofibrillary tangles were mildly attenuated in ALE 100. However, the significant lipid peroxidation was retained in ALE 100 along with development of partially degenerated or condensed neurons (Figure 10). The histological examination of ALE 200 and ALE 300 brain sections revealed tissue with healthy neurons, devoid of cytoplasmic vacuolations and neurofibrillary tangles, but with some hyperchromatic neurons.

#### Acute Oral Toxicity

Results showed that *A. lebbeck* (L.) seed extract was safe at 2000 mg/kg dose, as no mortality was seen till the 14th day.

### 3.6. In-Silico Modeling

Anticholinesterase activity of ALE was further corroborated with in-silico modeling of its phytochemicals. The docking simulation indicated all the ligands with negative binding energies that served as a function of binding affinity towards AChE. Galantamine served as a standard with −5.8 Kcal/mol of binding energy (ΔG) and 0.056 mM Ki. Galantamine revealed the molecular binding pattern of interaction by H-bonding with TYR341, ARG269, and water molecules at the catalytic site. The parameters of binding simulation are tabulated for the best binding mode of all the ligands for AChE (Table 5).

**Figure 11:**
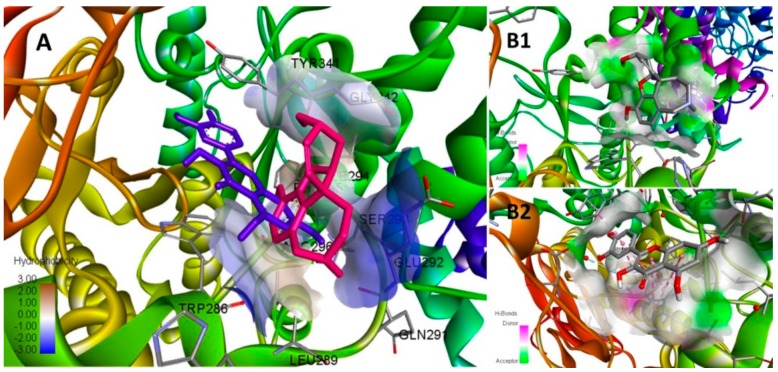
Conformational analysis of galantamine and quercetin docked at the catalytic site of AChE; (**A**) Simulated best binding mode of galantamine (pink) and quercetin (blue) shares the same binding pocket with quercetin oriented more towards the hydrophobic domain. (**B1**) Galantamine stabilized its conformation mainly via a pattern of hydrogen bonding colored as green (donor) and pink (acceptor) sites at the pocket. (**B2**) The conformation of quercetin is stabilized by the pattern of hydrophobic interactions (gray site) in addition to H-bonding at the pocket.

Quercetin revealed the highest binding affinity among all the ligands and exceeded the standard’s threshold with −8.3 Kcal/mol ΔG and 0.000824 mM Ki. The best binding mode of quercetin and galantamine are simulated for their pattern of hydrogen bonding at the catalytic site of AChE (Figure 11). The inhibition constant (Ki) of quercetin was followed by the Ki values of *m*-coumaric acid (0.066 mM), gallic acid (0.11 mM) and sinapic acid (0.13 mM) comparable to galantamine.

**Figure 12:**
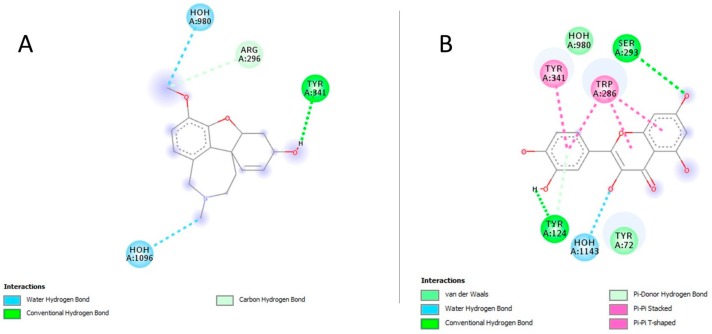
The potential interactions of galantamine and quercetin at the catalytic site of AChE; two-dimensional visualization of galantamine (**A**) and quercetin (**B**) interactions with key residues depicted as balls colored by the type of interactions.

Quercetin complex was stabilized by H-bonds with SER293 and TYR124, one π-π stacked bond with TYR341, three π-π stacked bonds with TRP286, and van der Waals interactions with TYR72 at catalytic site of enzyme (Figure 12). Quercetin and gallic acid shared the conserved interaction of galantamine with TYR341 at the active site.

## 4. Discussion

Plants have continuously retained their historical significance as a rich source of diverse, novel, and efficacious bioactive compounds for drug discovery and development. The medicinal potential of plants has inspired scientists across the board of scientific disciplines. Plants have been contributing milestones in situations where modern approaches (e.g., high-throughput screening) have failed. The present study exploits the hybrid of a traditional system of medicine and a random approach to use the seeds of *A. lebbeck* (L.) for AD therapeutics [30]. This study characterized the anti-AD potential of *A. lebbeck* seeds, spanning the phytochemical, behavioral, biochemical, histological, and computational modeling. Physicochemical analysis of the powder material revealed the acceptable hygroscopicity. The moisture content is critical to the stability and purity of the natural product as it facilitates the hydrolytic degradation and microbial contamination [31]. The selection of the solvent is a key determinant of extraction efficiency, the extract’s phytochemical composition, and purity [32]. These physicochemical results also indicated the efficiency of aqueous methanolic solvent to provide the good yield (i.e., 9.9%) with purity. Preliminary phytochemical analysis of powder material suggested a higher fraction of carbohydrates as compared to proteins and lipids. The *FTIR* analysis fingerprinted the phytochemicals with the presence functional groups of alkaloids, polyphenolics, flavonoids, and halogenated hydrocarbons [29]. Together, these analyses standardized the identity and quality of the plant material. Quantitative analysis of primary metabolites revealed the higher fraction of glycosaponins (68 ± 0.6%) in ALE that agreed with qualitative phytochemical analysis of its powder material. Consistently, solvent extractive efficiency was also manifested with higher fraction of flavonoids and polyphenolics in ALE. Flavonoids and polyphenolics are the bioactive secondary metabolites naturally endowed with immense therapeutic potential, specifically to fix the redox balance [33]. The antioxidant potential of these phenolic compounds depends upon the degree of structural substitution, hydroxylation, conjugation, and polymerization [34]. Plants have the innate ability to induce the endogenous antioxidant enzymes and scavenge the free radical corresponding to the diverse range of these polyphenolic metabolites [35]. In our present study, the higher content of these phenolic metabolites was consistent with the significant in vitro antioxidant potential of ALE as corroborated in DPPH and reducing power assay. The HPLC-UV analysis of ALE profiled these flavonoids and polyphenolics as quercetin, gallic acid, *m*-coumaric acid, and sinapic acid. Quercetin, a polyphenolic flavonoid, is an established potent antioxidant compound that has been reported to possess 3.5 times more total antioxidant capacity (TAC) as compared to curcumin [36]. Gallic acid, a phenolic tannin, is a versatile bioactive compound with antioxidant potential comparable to the α-tocopherol [37]. Sinapic acid, a phenolic acid, has been reported to inhibit the DPPH free radical (33.2%) equivalent to butylated hydroxytoluene (29.2%) and comparable to caffeic acid (49.6%) and α-tocopherol (41.8%) [38]. *m*-coumaric acid, a phenolic acid, constitutes as a main phytochemical of many plant species with potent antioxidant properties [39]. Together, all these phytochemical investigations validated the in vitro antioxidant potential of ALE. Toxicity studies showed the safety profile of the plant.

Oxidative stress has been the main hallmark of AD pathogenesis that precedes the ultrastructural neurological pathologies. The neuronal lipid bilayers are highly vulnerable to reactive species for lipid peroxidation that serves neurodegeneration in AD [40]. These free radicals have also been associated with protein oxidation and cross-linking, developing into cytoplasmic neurofibrillary tangles and senile plaques [41]. There is mounting evidence suggesting the efficacy of antioxidants in ameliorating neurodegenerative alterations in AD [42]. Therefore, this study was oriented to investigate the effectiveness of ALE in AD as supported by the context of its antioxidant properties and traditional uses. Aluminum chloride is a neurotoxic agent that preferably accumulates in the brain and induces the oxidative stress, memory, and cognitive deficits in the development of AD [43,44]. In this study, the experimental rats treated with aluminum chloride 100 mg/kg showed severe memory and cognitive deficits in behavioral tests. In the Morris water maze, AlCl_3_ rats displayed a decline in the capacity to process, retain, and retrieve the spatial information to locate the platform’s quadrant as depicted with lower swim time in it during the probe trial session. Simultaneously, ALE co-administration revealed the improvements in spatial learning and retention of long-term memory as reflected by increase in swim time for the same quest. This context-dependent memory model was further supported by the inclusion of associative memory models i.e., open field test and hole board. The enrichment of the environment enhances the tendency of exploration as a cognitive function of neophilia in normal rats [45]. In the open field test, ALE co-administration markedly improved the horizontal explorations and reversed the fear or anxious tendencies induced by aluminum chloride. The hole board model revealed the ALE co-administration significantly restored the neophillic response (i.e., head-dipping) of aluminum chloride-treated rats. However, consistent with previous studies, this exploratory tendency was diluted by the time in ALE treatment groups as hole board loosed its novelty by the development of associative memory [46,47,48]. These improvements in cognitive abilities and memory were further corroborated with function of working memory or short-term memory in Y-maze and T-maze tests. In accordance with previous studies, the degree of alternation (i.e., percentage of alternation) of the AlCl_3_ group was severely declined in Y-maze, which reflected a lower degree of learning and development of short-term working memory [20]. The AlCl_3_ group also revealed longer latency (s) to find the reward, i.e., rewarding alternation, which further extends support for the presence of short-term memory and cognition deficits [21]. However, the percentage of alternation and rewarding alternations was significantly improved with ALE co-administration, which was comparable to STD, and reflected the improved cognition and short-term working memory. Therefore, the improvements in spatial, associative, and working context of cognition and memory may foster the development of integrated cognitive maps that aid and restore the social interactions of life that have been previously depressed in AD [49,50].

These improved functional outcomes of behavioral studies were further supported by the biochemical estimation of pathological oxidative stress in AD. Aluminum has been implicated in the induction of redox-active iron concentration and depression of endogenous antioxidant enzyme system (e.g., SOD and CAT) in the brain [51]. In our study, we found a significant depletion of glutathione and marked depression of SOD and CAT activity in the brain tissue of the AlCl_3_ group in accordance to previous studies [23]. SOD has been implicated in the mitigation of oxidative stress by neutralizing the oxygen free radical (O_2_) into hydrogen peroxide (H_2_O_2_), which is further neutralized into water and oxygen by CAT enzymatic activity consuming GSH [52]. The depression of SOD and/or CAT activity results in cellular load of O_2_· and H_2_O_2_ that further, with redox-active iron (Fenton-type reaction), produces the more toxic hydroxyl (OH·) free radical, thereby inducing oxidative stress [53]. There has been evidence that flavonoid chelate and stabilize metal ions such as iron and aluminum [54]. The polyphenolics have been widely reported to ameliorate the progression of AD by mitigating oxidative stress [55]. Consistent to these studies, our results indicated that ALE co-administration significantly recovered the SOD and CAT activity along with induction of GSH content. These biochemical improvements complemented the experimental results of behavioral studies. The support to these biochemical outcomes was extended by the histological examinations of brain tissue that revealed the significant attenuation of lipid peroxidation, neurofibrillary tangles, and neurodegeneration with ALE co-administration.

In addition to neurodegeneration, loss of central cholinergic neurotransmission has been largely implicated as a pathological event of AD [56]. So far, AChE is the only therapeutic target for which the FDA has approved drugs to restore the cholinergic neurotransmission in AD. AChE is the primary hydrolytic enzyme responsible for degradation of acetylcholine (ACh) in synapses of cholinergic neurons [57]. Aluminum has been found to stimulate the activity of central AChE, resulting in the loss of cholinergic neurotransmission as manifested in the pathogenesis of AD [58]. Our study indicated the marked increase in activity of AChE following the aluminum chloride administration in AlCl_3_ that was in line with the literature [23]. Aluminum has been found to induce the structural modifications in AChE that enhance its catalytic potential [59]. However, ALE co-administration was found to significantly reverse the activity of AChE comparable to NC, which further lends support to the results of behavioral studies. This study was followed by docking simulations to further delineate the mechanism of ALE to modulate the activity of AChE. The docking studies provided insights into the ligand–AChE complex orientations and interactions to elucidate the inhibitory potential of ALE secondary metabolites based on the binding energy (ΔG) and inhibition constant (Ki) of this complex. All the secondary metabolites showed the inhibitory potential towards AChE as depicted by their affinity or binding energy, and Ki comparable to galantamine. This may justify the comparable inhibitory potential of ALE. Normally, drugs are considered to be effective with the Ki < 1mM [60]. As the exception among all the ligands, the inhibitory potential of quercetin was the highest, which exceeded the standard threshold of ΔG and Ki and may suggest it is the most active component among the secondary metabolites of ALE. Its superior inhibitory potential can be justified with its extensive and diverse interactions at the active site of AChE as compared to galantamine. In these simulations, it was reasonable to assume the relationship between the phenolic functionality and ΔG or Ki of phytochemicals, which may provide insights into novel pharmacophores for AChE inhibitors.

## 5. Conclusions

*Albizia lebbeck* seeds possessed two important antioxidants, quercetin and gallic acid. The data also suggest that the extract has high potential to significantly improve memory and cognition functions by inhibiting anticholinesterase, thereby preserving acetyl choline concentration, which is strongly linked to the integrity of memory and cognitive functions. Also, the computational modeling of its phytochemicals highlights the potential for bioactive molecules to fuel AD therapeutics.

## Figures and Tables

**Table 1:** Physicochemical analysis of powder (%).

Moisture Content	Water-Soluble Extractives	Alcohol-Soluble Extractives	Total Ash Content	Water-Insoluble Ash	Acid-Insoluble Ash	Sulphated Ash Content
8.5	7	4	11	91	18.18	40

**Table 2:** Phytochemical analysis of extract for primary and secondary metabolites in Percentage.

**Primary Metabolites**	Total protein content %	2.277 ± 0.007
	Total glycosaponins %	68.033 ± 0.606
**Secondary Metabolites**	Total alkaloids content %	1.27 ± 0.002
	Total polyphenolics %	88.6 ± 0.032
	Total flavonoids %	36.327 ± 0.049

Data are represented as Mean ± SD.

**Table 3:** Identification of the metabolites in HPLC analysis.

Peak	Retention Time (Min.)	% Area	Compounds
1	3.447	20.6	Quercetin
2	5.093	11.7	Gallic acid
4	20.333	6.9	*m*-Coumaric acid
5	25.927	5.9	Sinapic acid

**Table 4:** Estimation of endogenous antioxidant capacity of brain tissues of the experimental groups.

Groups	Treatment	DOSE (mg/kg)	GSH (µg/mg of Brain Tissue)	SOD (µg/mg of Brain Tissue)	CAT (µg/mg of Brain Tissue)
I	Normal control	N/A	79.26 ± 1.09	27.49 ± 0.35	23.34 ± 0.83
II	Disease control (AlCl_3_)	100	21.01 ± 0.89	12.05 ± 0.61	4.39 ± 0.53
III	Standard (STD)	0.8	79.68 ± 2.27 ***	24.68 ± 1.76 ***	12.46 ± 1.09 ***
IV	ALE 100	100	65.56 ± 3.58 ***	23.82 ± 0.98 ***	9.64 ± 1.65 *
V	ALE 200	200	76.56 ± 1.06 ***	24.73 ± 0.25 ***	9.85 ± 0.67 *
VI	ALE 300	300	80.14 ± 2.55 ***	26.43 ± 0.68 ***	22.60 ±0.62 ***

*** *P* < 0.001 (highly significant) and ** *P* < 0.001 as compare to disease group. Each value is mean ± SEM.

**Table 5:** Parameters of binding simulation against AChE.

Compound	Binding Energy (Kcal/mol)	Ki (mM)	Interacting Residues	Interaction Types
Galantamine	−5.8	0.056	TYR341, ARG269	H-Bonding
Quercetin	−8.3	0.000824	TYR341, TRP286, SER293, TYR124, TYR72	H-Bonding, π-π Stacked, van der Waals
*m*-Coumaric acid	−5.7	0.066	LEU540, PRO537, LEU536, ASN533	H-bonding, π-Alkyl
Gallic acid	−5.4	0.11	TYR341, SER293	H-Bonding
Sinapic acid	−5.3	0.13	ASP349, SER347, GLY345, VAL343, GLY342	H-bonding
